# Effectiveness of conventional versus virtual reality based vestibular rehabilitation in the treatment of dizziness, gait and balance impairment in adults with unilateral peripheral vestibular loss: a randomised controlled trial

**DOI:** 10.1186/1472-6815-12-3

**Published:** 2012-03-26

**Authors:** Dara Meldrum, Susan Herdman, Roisin Moloney, Deirdre Murray, Douglas Duffy, Kareena Malone, Helen French, Stephen Hone, Ronan Conroy, Rory McConn-Walsh

**Affiliations:** 1 Royal College of Surgeons in Ireland, 123 St. Stephen's Green, Dublin 2, Ireland; 2 Department of Rehabilitation Medicine, School of Medicine, Emory University, Atlanta, USA; 3 Beaumont Hospital, Dublin 9, Ireland; 4 Royal Victoria Eye and Ear Hospital, Adelaide Road, Dublin 2, Ireland

**Keywords:** Rehabilitation, Vestibular diseases, Nintendo Wii Fit Plus^®^, Virtual reality, Postural balance, Dizziness, Vertigo, Gait, Visual acuity, Feedback sensory

## Abstract

**Background:**

Unilateral peripheral vestibular loss results in gait and balance impairment, dizziness and oscillopsia. Vestibular rehabilitation benefits patients but optimal treatment remains unkown. Virtual reality is an emerging tool in rehabilitation and provides opportunities to improve both outcomes and patient satisfaction with treatment. The Nintendo Wii Fit Plus^® ^(NWFP) is a low cost virtual reality system that challenges balance and provides visual and auditory feedback. It may augment the motor learning that is required to improve balance and gait, but no trials to date have investigated efficacy.

**Methods/Design:**

In a single (assessor) blind, two centre randomised controlled superiority trial, 80 patients with unilateral peripheral vestibular loss will be randomised to either conventional or virtual reality based (NWFP) vestibular rehabilitation for 6 weeks. The primary outcome measure is gait speed (measured with three dimensional gait analysis). Secondary outcomes include computerised posturography, dynamic visual acuity, and validated questionnaires on dizziness, confidence and anxiety/depression. Outcome will be assessed post treatment (8 weeks) and at 6 months.

**Discussion:**

Advances in the gaming industry have allowed mass production of highly sophisticated low cost virtual reality systems that incorporate technology previously not accessible to most therapists and patients. Importantly, they are not confined to rehabilitation departments, can be used at home and provide an accurate record of adherence to exercise. The benefits of providing augmented feedback, increasing intensity of exercise and accurately measuring adherence may improve conventional vestibular rehabilitation but efficacy must first be demonstrated.

**Trial registration:**

Clinical trials.gov identifier: NCT01442623

## Background

Unilateral peripheral vestibular loss (UVL) results in disabling problems including vertigo, dizziness, oscillopsia, and impaired balance and gait [1,2]. In the acute phase most patients are managed at primary care level and recover through a process known as vestibular compensation [3]. Pharmacological treatments are recommended only in the acute phase [4] and the mainstay for treatment thereafter in those who do not recover, is specialised rehabilitation known as vestibular rehabilitation. Vestibular rehabilitation is a safe, effective and non-invasive treatment for the sequelae of UVL [5,6]. Subjectively, balance impairment is rated as more problematic than dizziness or vertigo [7] and while meta-analysis has demonstrated a clear benefit for the reduction in dizziness and vertigo symptoms, a less convincing effect has been shown for reducing balance and gait impairment [5,8]. Most studies have measured the outcome of vestibular rehabilitation on subjective symptoms, static balance, and visual acuity during head movement and but have not measured in any detail its effect on gait impairment. Galvanic vestibular stimulation studies have shown that the vestibular system plays a phase dependent role in gait and is active at critical points in the gait cycle, particularly during double support, changing direction, and step termination [9]. Bent et al [9] concluded that "rehabilitation strategies should focus on compensating for deficient vestibular inputs during the gait cylce".

Rehabilitation of gait and balance during a vestibular rehabilitation program requires motor learning and thus practice and feedback. In conventional rehabilitation it is difficult for patients to gain feedback when performing balance exercises which can be repetitive and boring. Force plate technology has been used in the clinical setting to provide visual and auditory feedback during balance re-education and has shown some promising results [10,11]. Virtual reality, defined as a high-end-computer interface that involves real time simulation and interactions through multiple sensorial channels' [12] is also being investigated in laboratory settings [13]. Virtual reality provides feedback and also allows a more stimulating and enriching environment than usual rehabilitation. It can measure and track exercise, and allows virtual experience of activities the patient might otherwise not attempt [14].

**Figure 1:**
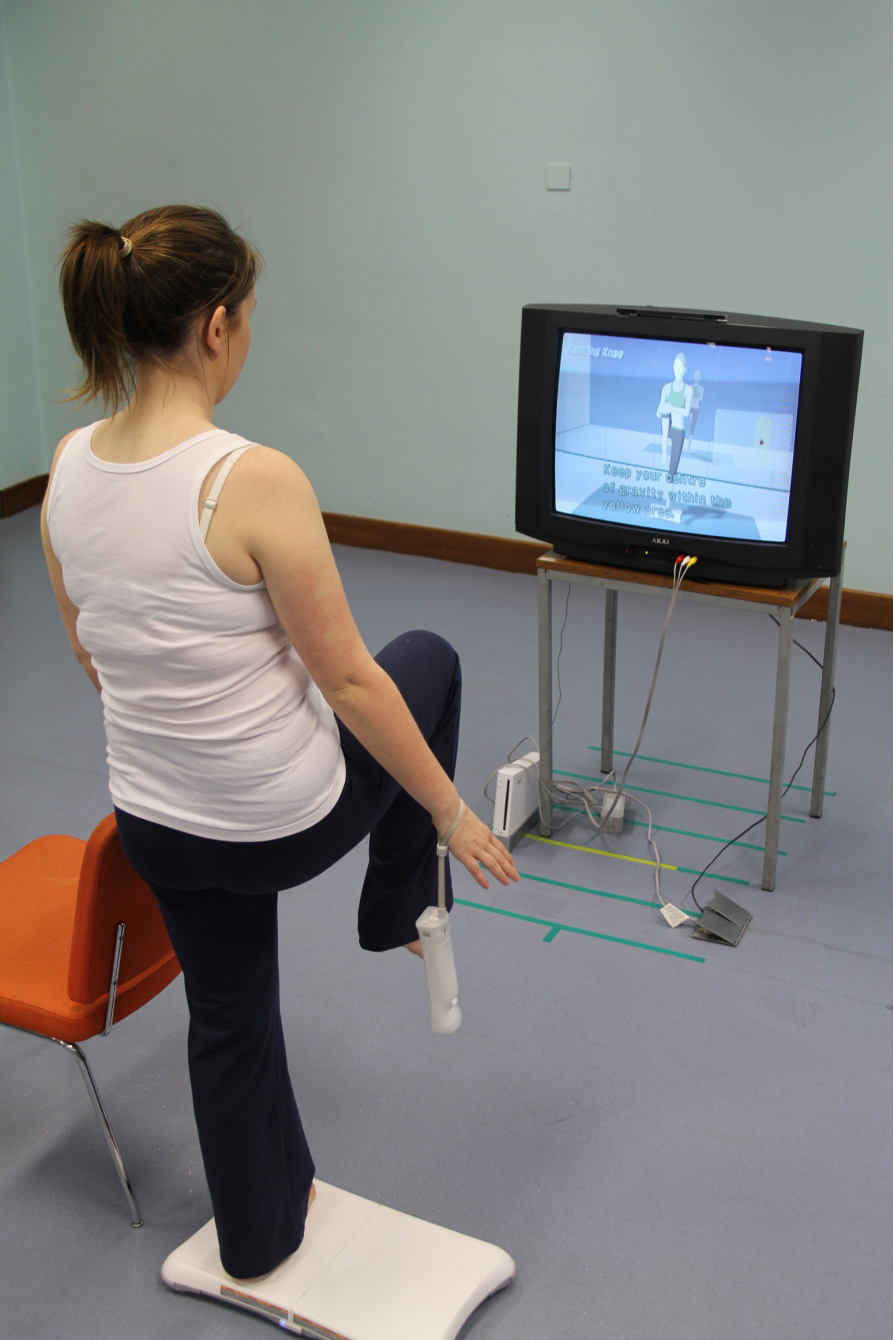
**The Nintendo Wii Fit Plus^®^**.

There is some support for the use of virtual reality in vestibular rehabilitation [15] but most technologies are presently prohibitively expensive, considered to be for research use, and are not universally available. They require considerable therapist time and are unlikely to be used with the frequency that is required for motor learning, particularly in vestibular rehabilitation where most programs entail daily exercise. Recent developments in the gaming industry have resulted in the Nintendo Wii^® ^Fit Plus (NWFP, Figure 1). The NWFP combines an accelerometer and a forceplate to provide visual and auditory feedback of subjects' centre of pressure during virtual reality exercises and games. It stimulates movement and perturbs balance in order to retrain it. Use of this low cost gaming system could easily be adapted to meet the requirements of a vestibular rehabilitation program. The system allows for accurate monitoring of use (intensity, type and frequency) which is an important area that has proven difficult to monitor in research and clinical settings due to inaccurate patient recall or records. It is also fun to use and therefore motivating for the patient.

**The Nintendo Wii Fit Plus^®^**. The participant stands on the Wii balance board which monitors centre of pressure and reconstructs it visually on the television monitor as a red dot within a yellow area. A variety of exercises and games are available to retrain balance and encourage head movement. Stepping exercises can also be performed encouraging stopping and changing direction, in which the vestibular system has a role. Written consent to reproduce the image was provided by the subject in the photo.

The rehabilitation community is beginning to investigate the NWFP in the area of balance retraining [16,17]. Anecdotal reports indicate the NWFP is being used in vestibular rehabilitation [18] but as yet no randomised controlled trials exist. Importantly, Clark et al [19] recently showed that the NWFP force plate (Wii Board) tracks the centre of pressure with a similar accuracy to a gold standard forceplate. This provided initial support for the use of the NWFP in rehabilitation. Additionally we have shown that patients with vestibular problems found the NWFP highly usable, enjoyable and the majority of them preferred it to conventional balance re-education [20]. As yet no study has investigated the efficacy of the NWFP in vestibular rehabilitation. The aim of this study is to compare the outcome from conventional vestibular rehabilitation to virtual reality based vestibular rehabilitation using the Nintendo Wii Fit Plus^®^.

### Pilot study

As there are no reports of Nintendo Wii^® ^use in vestibular rehabilitation, a pilot study was carried out to investigate patient experiences with the NWFP^® ^[20]. The study found high levels of usability, acceptance and enjoyment of the NWFP^® ^as a therapy. The study assessed 26 participants with balance impairment (14 of whom had vestibular disease) performing NWFP^® ^exercises and games that will be incorporated as part of the proposed study and evaluated safety andsymptoms provoked. The average verbal rating scale for enjoyment (with higher scores indicating more enjoyment) was 8/10. The average System Usability Score [21] was 82% indicating high usability (defined as the learnability, ease of use and memorability of a system). Most (88%) participants reported they would like to use it in future treatment; 67% reported more enjoyment and motivation than conventional therapy. There were no significant adverse effects but for safety purposes patients required additional haptic support during exercise (in the form of a chair) [20].

## Methods/Design

**Figure 2:**
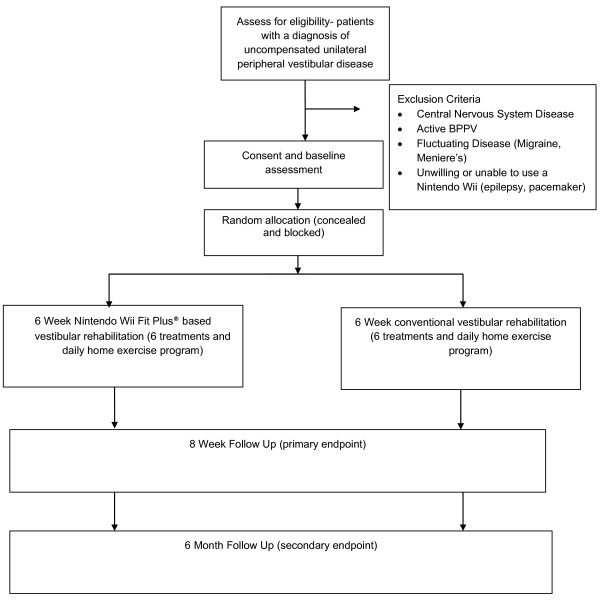
**CONSORT (2010) flow diagram of the trial**.

The study protocol has been designed with reference to the CONSORT statement in order to ensure methodological validity [22]. It is not possible to blind treating therapists nor patients to the treatment intervention so a single (assessor) blind, randomised controlled trial design will be used. It is also considered unethical to have a "no treatment" or a placebo control group as treatment for peripheral vestibular dysfunction has a robust evidence base [5]. The aim of this study is to compare the outcome from conventional vestibular rehabilitation to virtual reality based vestibular rehabilitation in the treatment of peripheral vestibular impairment. A parallel design will be used. Figure 2 shows the flow of diagram of the trial.

Study objectives

1. To compare the effect of conventional vestibular rehabilitation and virtual reality based rehabilitation on gait impairment.

2. To compare the effect of conventional vestibular rehabilitation and virtual reality based rehabilitation on balance.

3. To compare the effect of conventional vestibular rehabilitation and virtual reality based rehabilitation on dizziness and vertigo.

4. To compare the effect of conventional vestibular rehabilitation and virtual reality based vestibular rehabilitation on dynamic visual acuity.

5. To quantify and compare patient satisfaction and adherence with conventional and virtual reality based vestibular rehabilitation.

### Participants and setting

Patients attending the otolaryngology and neurology outpatient clinics in two Dublin university teaching hospitals (Beaumont Hospital, Dublin and the Royal Victoria Eye and Ear Hospital, Dublin) for diagnosis and treatment of unilateral vestibular loss will be invited to participate in the trial. Participants will be provided with information leaflets and given a cooling off period to consider their involvement in the trial. Written informed consent will be obtained and patients will be free to withdraw from the study at any time. Non-consenting participants will be treated with usual care.

### Inclusion criteria

Participants will be included if they have a clinical diagnosis of peripheral vestibular dysfunction (confirmed where possible with vestibular function testing; canal paresis > 20%), and no other neurological deficit. Caloric testing is not available to all patients in the sites; where it is not available presence of a positive head thrust test, head shaking after nystagmus or spontaneous nystagmus (assessed with infrared oculomotor recording system) will be used to support the clinical diagnosis. Participants will also have one of the following subjective complaints indicating a failure of vestibular compensation; dysequilibrium, gait instability, vertigo/dizziness, or motion sensitivity.

### Exclusion criteria

Participants will be excluded if they have had previous vestibular rehabilitation or have bilateral peripheral vestibular pathology, CNS involvement, fluctuating symptoms Meniere's disease, migrainous vertigo), active benign paroxysmal positional vertigo, or other medical conditions in the acute phase (orthopaedic injury). As per Nintendo Wii^® ^guidelines, participants will also be excluded if they have a pacemaker or epilepsy, or are unwilling or unable to use a NWFP. Pregnant patients will also be excluded.

### Randomisation procedure

A permuted blocked randomisation procedure will be used. A third party (HF) not involved in the day to day running of the trial will use an online randomisation program (http://www.randomization.com) to assign patients to either conventional therapy or NWFP therapy in advance of recruitment. Block size will be chosen randomly from a block size of 4, 6 or 8 and block size will not be revealed to other study personnel. Group allocation will be performed by the third party and notified to the treating therapist by email after individual patients provide informed consent and undergo baseline assessments (by the blinded assessor), thus ensuring concealed allocation.

### Outcome measures

Consenting participants will complete baseline assessments prior to randomisation. They will be retested post treatment (at 8 weeks). The primary endpoint is gait velocity (m/sec) at 8 weeks post baseline.

Gait velocity will be assessed using three dimensional gait analysis (3DGA) in the Royal College of Surgeons in Ireland, School of Physiotherapy's Movement Laboratory. The laboratory has a Vicon 250 motion analysis system (Vicon Motion Systems Ltd., Oxford, UK) and a Kistler force plate (Kistler Instruments Ltd., Winterthur, Switzerland). Vicon consists of five infra red cameras that are able to localize reflective markers (placed on the participant) in a three-dimensional coordinate system within 1.5 mm error. Because of its high level of accuracy and reliability, it is considered a "gold standard" for motion analysis. The Kistler force plate, in synchronicity with the Vicon is able to compute kinetic forces during gait as well as centre of mass and centre of pressure. Gait velocity will be recorded during four conditions; self selected speed eyes open, self selected speed eyes closed (towards a target), cadence of 120 steps per minute eyes open (paced with a metronome), and walking with head turns.

Secondary gait variables will also be investigated; percentage of a gait cycle spent in double support, base of support width (cm), centre of mass/centre of pressure moment arm (mediolateral) and centre of mass velocity and displacement. These variables have been identified by Krebs et al (2003) [8] as the most significant discriminators of success following vestibular rehabilitation The Dynamic Gait Index (DGI)[23] will also be assessed.

Other secondary outcomes will be;

1. Computerised dynamic posturography (Equitest-NeuroCom) [24]. This measures postural sway during six sensory conditions and returns sensory ratios that can be compared to normative data. The sensory organisation test (SOT) will be performed according to the protocol published by Equitest (Neurocom).

2. The Vestibular Rehabilitation Benefits Questionnaire (VRBQ) [25,26] and the Activities Specific Balance Confidence Scale (ABC) [27]. These are validated and reliable questionnaires for assessment of the effects of dizziness. Scores range from 0-100.

3. Dynamic Visual Acuity (DVA) [28]. This will be measured using a computerised DVA test (Micromedical Technologies, Chatham, Illinois). The difference between static and dynamic visual acuity (at three head speeds) in LogMAR units will be measured using the protocol published by Micromedical Technologies.

4. The Hospital Anxiety and Depression Scale (HAD) [29]. This validated scale has been used previously in studies in vestibular rehabilitation and assesses non-somatic symptoms of anxiety and depression. Scores range between 0 and 21, scores between 8 and 10 are considered borderline and those above ten indicate clinical depression or anxiety.

5. Usability of virtual reality technology will be measured by the System Usability Questionnaire [21]. This is a 10 item questionnaire using Likert scales. Adherence will be recorded in the exercise booklets (and where allocated, on the NWFP) and satisfaction with treatment will be measured with a questionnaire developed by the researchers.

A six month follow up will be carried out. This will be confined to the primary outcome measure of gait velocity (m/sec), and secondary outcomes of computerised posturography, the VRBQ, ABC and DGI. Outcome measurement at each time point is shown in Table 1.

**Table 1:** Time frame and outcome measures in the study

Outcome Measure	Baseline Assessment	Post treatment (8 weeks)	Post Treatment (6 months)
Gait Speed	**✓**	**✓**	**✓**
Dynamic Gait Index	**✓**	**✓**	**✓**
Dynamic Posturography	**✓**	**✓**	**✓**
Activities Balance Confidence Questionnaire	**✓**	**✓**	**✓**
Vestibular Rehabilitation Benefits Questionnaire	**✓**	**✓**	**✓**
Dynamic Visual Acuity	**✓**	**✓**	
Hospital Anxiety and Depression Scale	**✓**	**✓**	
Adherence/Satisfaction with treatment			
*Three Dimensional Gait Analysis*			
Centre of mass velocity and excursion	**✓**	**✓**	
Centre of mass/centre of pressure moment arm	**✓**	**✓**	
% gait cycle in double support	**✓**	**✓**	
step width (cm)	**✓**	**✓**	

### Sample size and power calculation

Based on previously published work the estimated sample sizes for each outcome variable have been calculated (Table 2). The calculations were performed to detect what would be a clinically important difference between the means, with a significance level of 0.05 using the power calculation by Pocock [30] given as;

**Table 2:** Sample size and power calculations for the trial

Outcome Measure	Mean Change Expected	SD	Sample Size Required (per group)
Gait Velocity (m/sec)	0.1	0.15	36
Vestibular Rehabilitation Benefit	-7	10.1	33
Questionnaire (%) [25]			
Static Posturography (% composite score)[31]	10	15	36
Dynamic Visual Acuity (LogMAR) [32]	0.1	.136	30

$$\text{n}=\frac{2\sigma^{2}}{{\left(\mu_{2}-\mu_{1}\right)}^{2}}\times \text{f}(\alpha ,\beta )$$

Where σ is the standard deviation, μ is the mean difference expected between the two groups using a two sample t-test, α is the significance level (P < 0.05) and β is the lower (0.80) and f (α,β) is 7.9. Allowing for a 5% drop-out rate a minimum of 37 patients will be required pergroup. Current referral rates of patients with unilateral vestibular loss to physiotherapy approximate 40-50 participants per year over the two sites, thus a two year recruitment period is required.

### Interventions

Following baseline assessments (by the blinded assessor), participants will be randomised (as described above) to one of two groups;

1. Conventional vestibular rehabilitation

2. Virtual reality vestibular rehabilitation

Based on best available evidence [5,6] 6 treatments will be provided over an 8 week timeframe. Treatment will only be provided by physiotherapists who are at senior level and have experience in vestibular rehabilitation and all treating physiotherapists will attend periodic protocol training sessions. On their first attendance participants will undergo a standardised physiotherapy assessment which will elucidate their main problems. The interventions for both groups have been developed and will be based on six identified core elements of vestibular rehabilitation used in current clinical practice- education, relaxation, adaptation exercises, habituation exercises, balance and gait retraining and re-conditioning [33]. Programs are customised to symptoms and impairments, and are progressive. All participants will be asked to perform a home exercise program daily for 30-40 minutes. Each participant will receive a standardised weekly booklet detailing their exercises and containing an adherence diary. They will return the booklets at each treatment session and receive a new one. The booklets have been designed to look similar. Each is tripartite containing adaptation exercises, balance exercises and a walking program. The booklets, provided to both groups differ only in the balance exercises, the conventional group receiving conventional balance exercises [2,34] and the NWFP group receiving exercises developed in pilot work [20]. The NWFP records type, duration and frequency of exercises and this will be used as an additional record in the NWFP group.

Participants in the virtual reality group will be instructed in the use of the NWFP^® ^and will be given one on loan for use at home. Participants in the conventional group will be provided with a standard therapeutic foam balance mat (Sissel Balance Fit Pad). All patients will be reviewed a minimum of 4 times over 8 weeks for re-assessment, progression of exercises and advice by the treating therapist.

### Safety considerations

To address the risk of falling, participants will be provided with the necessary written and verbal instructions to prevent a fall (e.g. support nearby). Any adverse events will be reported to the participants consultant physician and an adverse event form filled out. Participants experiencing adverse events will be followed up and assessed by their treating physiotherapist and physician.

#### Data analysis

Data will be coded and entered in Microsoft Excel and statistical analysis will be performed using PASW 18 (SPSS Inc. Chicago) and Stata Statistical Software (Release 11, StataCorp LP, TX:). Intention to treat analysis and per protocol analyses will be performed [34]. Baseline data will be examined for comparability. Where there are missing data, the last observation will be carried forward. Data will be summarised using means, standard deviations and 95% confidence intervals for continuous variables, median and interquartile ranges for non-normal continuous or ordinal data and percentages for categorical data. Per-protocol analyses will be performed excluding patients with major deviations from the treatment protocol (defined as less than 50% adherence with treatment). Data will be examined for normality and t-tests used for data with two time points. A mixed ANOVA model will be used for data with three time points with the factors of group (NWFP or Conventional Group) and time (baseline, 8 weeks, six months). Within and between group differences and interaction effects will be calculated and post hoc analyses performed where indicated. Comparison of outcome measures between groups will be performed controlling of the baseline distribution of known predictor variables and the results will be reported as differences in means/medians and their confidence intervals [32,35]. The non-parametric equivalent will be used where data are not normally distributed or are non-parametric. A significance level of p < 0.05 will be set. Effect sizes for within and between groups comparison will also be calculated.

### Ethics

Ethical approval has been obtained from the Beaumont Hospital (Ref 10/75) and the Royal Victoria Eye and Ear Hospital medical ethics committees (Ref 24/10/2011). Participants will receive a verbal explanation of the study from the principle investigator (DM), will be given an opportunity to ask questions and will receive an information sheet. A cooling off period of a time designated by the participant will be given. Written informed consent will be obtained. Participants will be informed that they may leave the study at any time without a need to give an explanation. The study will be carried out in accordance with the Declaration of Helsinki Guidelines.

## Discussion

This protocol sets out the process by which we intend to investigate whether vestibular rehabilitation utilising the Nintendo Wii Fit Plus^® ^virtual reality system is more effective than conventional rehabilitation of unilateral peripheral vestibular loss. Based on the theoretical knowledge that feedback and practice improve motor learning [36], incorporating feedback technology such as virtual reality in rehabilitation programmes presents therapists with considerable opportunities to improve patient outcomes.

Of particular importance are the effects on gait and balance as optimal treatment and the mechanisms by which these improve presently lack sufficient knowledge and evidence. In combination with the visual and auditory feedback that the NWFP provides, other aspects such as enjoyment, challenge, an enriched sensory environment and the subsequent effect on adherence also require consideration. These issues will be addressed in the current study and will provide some knowledge on how individuals with unilateral peripheral vestibular loss adhere to and interact with virtual reality programs.

Rehabilitation is often criticised for lack of standardisation of treatment and this study will standardise the frequency, type and intensity of treatment across both groups in order to allow for valid comparisons.

The results of this study will provide information on the efficacy the NWFP in vestibular rehabilitation and will assist therapists in making treatment decisions when treating patients with unilateral peripheral vestibular loss.

## Competing interests

The authors declare that they have no competing interests.

## Authors' contributions

All authors participated in the conception and design of the trial. Dara Meldrum, Susan Herdman and Rory McConn Walsh designed the study and secured the funding. Dara Meldrum and Susan Herdman formulated the interventions for the study. Helen French drew up and performed the randomization and concealment of allocation plan. Ronan Conroy advised on the statistical analysis plan. Roisin Moloney, Kareena Malone, Deirdre Murray and Douglas Duffy are treating physiotherapists in the study and critically evaluated and contributed to the design of the interventions. Stephen Hone critically read and commented on the study protocol. All authors read and approved the final manuscript.

## Pre-publication history

The pre-publication history for this paper can be accessed here:

http://www.biomedcentral.com/1472-6815/12/3/prepub
